# Effects of a multiple chronic condition (CC) remote monitoring program on clinical outcomes among older adults

**DOI:** 10.1093/geroni/igab046.3686

**Published:** 2021-12-17

**Authors:** Eldin Dzubur, Roberta James, Jessica Yu, Julia Hoffman, Bimal Shah

**Affiliations:** 1 Teladoc Health, pasadena, California, United States; 2 Teladoc Health, Mountain View, California, United States

## Abstract

Older adults are faced with an increased risk of comorbid chronic disease such as diabetes. While multiple health behavior change interventions (MHCIs) are known to improve clinical outcomes more than targeted interventions, less is known whether such effects persist in older populations. The objective of the study was to examine the effects of multiple chronic condition (CC) remote monitoring program enrollment and mental health program enrollment on glucose and blood pressure reduction, adjusting for self-monitoring behaviors. In a sample of 594 older adults (age 55+, 14% 65+ years, 46.8% female) evaluated over a 12-month period, statistical models showed that older adults with uncontrolled diabetes (A1c >= 7.0%) had a 7.9 pt. reduction in blood glucose for each additional program enrolled and a 22.7 pt. reduction in blood glucose when enrolled in mental health compared to those not enrolled. Similarly, older adults with uncontrolled hypertension (BP >= 130/80) had a 4.8 pt. reduction in systolic blood pressure for each additional program enrolled and a 7.2 pt. reduction in systolic blood pressure when enrolled in mental health compared to those not enrolled. The findings indicate the potential for multiprogram digital health interventions that incorporate mental health to further improve clinical outcomes in older adults suffering from multiple chronic diseases, namely diabetes and hypertension.

